# Immunotherapies for postural orthostatic tachycardia syndrome, other common autonomic disorders, and Long COVID: current state and future direction

**DOI:** 10.3389/fcimb.2025.1647203

**Published:** 2025-09-29

**Authors:** Svetlana Blitshteyn, Gabriela Funez-dePagnier, Anna Szombathy, Meagan Hutchinson

**Affiliations:** ^1^ Department of Neurology, University of Buffalo Jacobs School of Medicine and Biomedical Sciences, Buffalo, NY, United States; ^2^ Dysautonomia Clinic, Williamsville, NY, United States; ^3^ University at Buffalo Jacobs School of Medicine and Biomedical Sciences, Buffalo, NY, United States

**Keywords:** postural orthostatic tachycardia syndrome, dysautonomia, autonomic disorders, immunotherapy, immunoglobulin, autoimmunity, therapeutics

## Abstract

Postural orthostatic tachycardia syndrome (POTS), neurocardiogenic syncope, and orthostatic hypotension are the most common autonomic disorders encountered in clinical practice. The autoimmune etiology and association of these conditions with systemic autoimmune and inflammatory disorders, autonomic neuropathy, and post-acute infectious syndromes, including Long COVID, suggest that immunotherapies should be considered as a therapeutic option, at least in a subset of patients. However, the treatment of common autonomic disorders has traditionally included pharmacologic and non-pharmacologic symptomatic therapies as the standard approach. Unfortunately, these symptomatic therapies have been of limited or insufficient efficacy to meaningfully improve functional status or result in recovery, especially in patients with severe symptoms. Case reports, case series, and clinical experience suggest that intravenous and subcutaneous immunoglobulin, as well as other immunologic therapies (such as plasmapheresis, corticosteroids, and rituximab), may be effective in some patients with severe POTS and other common autonomic disorders who are refractory to standard therapies. In this narrative review, we summarize the literature available on the topic of immunotherapies for POTS, other common autonomic disorders, and Long COVID. We also highlight the need for large, multicenter, placebo-controlled trials of immunoglobulin, plasmapheresis, intermittent corticosteroids, and other repurposed immunotherapies in patients with common autonomic disorders who have significant functional impairment.

## 1 Introduction

Postural orthostatic tachycardia syndrome (POTS), one of the most common disorders affecting the autonomic nervous system, is a disabling condition with no U.S. Food and Drug Administration (FDA)-approved treatment. Neurocardiogenic syncope, orthostatic hypotension, inappropriate sinus tachycardia, and post-COVID dysautonomia are other common autonomic disorders (OCADs) frequently encountered in clinical practice. The treatment of these conditions traditionally includes non-pharmacologic and pharmacologic regimens consisting of symptomatic treatment, which is currently accepted as the standard of care. However, for many patients with POTS and OCADs, these symptomatic therapies have been of limited and often insufficient efficacy, resulting in significant improvement or recovery. Case reports, case series, and clinical experience suggest that immunotherapies and immunomodulating agents may present potentially effective therapeutic options for some patients with standard treatment-refractory POTS and OCADs. In this narrative review, we discuss the available literature on the use of immunotherapies in POTS and OCADs, including post-COVID dysautonomia as part of Long COVID, and we discuss the complexities, challenges, and future direction of immunologic therapies as treatments for the underlying autoimmune and immune-mediated etiologies of these disorders.

### 1.1 Postural orthostatic tachycardia syndrome

POTS is a chronic disorder of the autonomic nervous system characterized by orthostatic tachycardia, which is defined as an increase in heart rate by ≥30 bpm in adults and ≥40 bpm in adolescents 12–19 years old, from supine to standing position, associated with orthostatic symptoms that last for at least 3 months (Freeman et al., 2011; Sheldon et al., 2015) (**Table 1**). Although it is defined by postural tachycardia, the clinical features of POTS are numerous and include dizziness, headache, fatigue, nausea, generalized weakness, and sleep disturbances (Low et al., 1995; Thieben et al., 2007). The pathophysiologic mechanisms of POTS are also numerous and diverse, including autoimmunity, hypovolemia, hyperadrenergic state, cerebral hypoperfusion, and small fiber neuropathy (Low et al., 1995; Thieben et al., 2007; Shaw et al., 2019). The onset of POTS may be sudden or insidious and can follow various triggers, such as infection, puberty, pregnancy, vaccinations, surgery, concussion, and injury (Shaw et al., 2019). Importantly, patients with POTS have diminished quality of life and functional impairment similar to patients with congestive heart failure and chronic obstructive pulmonary disease, with greater than 50% of patients unable to maintain employment (Benrud-Larson et al., 2002; Bourne et al., 2021).

**Table 1:** Diagnostic criteria for common autonomic disorders and Long COVID. .

Disorder	Diagnostic criteria	Clinical features
POTS (Freeman et al., 2011; Sheldon et al., 2015)	1. HR increase ≥30 bpm within 10 min for adults (≥40 bpm for adolescents 12–19 years of age) of standing or TTT. 2. Absence of OH, a ≥20 mmHg drop in systolic blood pressure. 3. Symptoms of orthostatic intolerance for ≥3 months.	Palpitations, exercise intolerance, dyspnea, tachycardia, chest discomfort, syncope, tremors, anxiety, blurred vision, headaches, lightheadedness, fatigue, weakness, gastroparesis (abdominal pain, nausea, and Irritable bowel syndrome (IBS)), and bladder dysfunction.
NCS (Freeman et al., 2011; Sheldon et al., 2015)	1. Transient loss of consciousness typically preceded by prodromal symptoms and signs. 2. A sudden fall in blood pressure, heart rate, and cerebral hypoperfusion on standing or TTT.	Prodromal symptoms may include pallor, diaphoresis, nausea, headache, and weakness. Loss of consciousness is typically brief and is not usually followed by confusion.
OH (Freeman et al., 2011)	Sustained drop in blood pressure ≥20/10 mmHg within 3 min of standing or TTT.	Syncope, presyncope, and dizziness.
IST (Freeman et al., 2011; Sheldon et al., 2015)	1. Average sinus HR exceeding 90 bpm over 24 h or HR while awake and at rest ≥100 bpm. 2. Palpitations and other distressing symptoms associated with sinus tachycardia.	Palpitations, dyspnea, lightheadedness, chest discomfort, and transient loss of consciousness.
Long COVID (Ely et al., 2024; National Academies of Sciences, E. and Medicine, 2024)	Symptoms that persist >12 weeks after probable or confirmed SARS-CoV-2 infection and last at least 2 months with no other culpable etiology.	Fatigue, shortness of breath, exercise intolerance, “brain fog”, headache, palpitations, loss of smell, poor memory, dizziness, altered mood, and sleep disturbance.

POTS, postural orthostatic tachycardia syndrome; NCS, neurocardiogenic syncope; OH, orthostatic hypotension; HR, heart rate; bpm, beats per minute; TTT, tilt table test; IST, inappropriate sinus tachycardia.

Prior to the COVID-19 pandemic, POTS was estimated to affect approximately 0.2%–1% of the US population (1–3 million people) (Vernino et al., 2021). After the COVID-19 pandemic, the incidence of POTS was found to have increased 15-fold due to POTS and autonomic dysfunction being common manifestations of Long COVID (Dulal et al., 2025). POTS predominantly affects women of reproductive age, ages of 15–25 (Vernino et al., 2021), but men are also becoming increasingly affected due to post-COVID POTS. Common comorbidities include migraines (at least 40%), gastrointestinal disorders (at least 30%), small fiber neuropathy (at least 50%), Ehlers–Danlos syndrome and hypermobility spectrum disorders (HSDs) (at least 30%), autoimmune disorders (at least 20%), myalgic encephalomyelitis/chronic fatigue syndrome (ME/CFS) (at least 20%), and mast cell activation syndrome (at least 20%) (Shaw et al., 2019).

There are no FDA-approved therapies for POTS, but a commonly accepted therapeutic approach to POTS consists of non-pharmacologic and pharmacologic treatment options. Pharmacotherapy includes first-line medications such as beta-blockers, which decrease resting and postural tachycardia by reducing sympathetic overactivity; fludrocortisone, a mineralocorticoid that augments retention of water and sodium and expands plasma volume; midodrine, which is an alpha-1 agonist that causes vasoconstriction and increased peripheral resistance; and pyridostigmine, a parasympathetic nervous system enhancer (Raj et al., 2022; Grubb and Grubb, 2023).

### 1.2 Neurocardiogenic syncope

Neurocardiogenic syncope (NCS) (also known as vasovagal syncope or neurally mediated syncope) is defined as a sudden fall in blood pressure, heart rate, and cerebral hypoperfusion on standing or a tilt table test (Freeman et al., 2011; Sheldon et al., 2015) (**Table 1**). It is usually of rapid onset and short duration and may be preceded by prodromal symptoms, such as pallor, diaphoresis, nausea, headache, and weakness. The loss of consciousness is typically brief and is not usually followed by confusion. NCS can occur after various triggers, including standing, pain, dehydration, heat, and the sight of blood. This form of syncope is common, with 42% of women and 32% of men experiencing at least one episode by age 60. Although when NCS occurs occasionally it is benign, recurrent and frequent NCS can greatly impair quality of life (Sheldon et al., 2015). One common mechanism of syncope involves ineffective reflex response, where baroreceptors fail to perceive drops in venous return upon standing or pathologic vasodilation is triggered. The resulting hypotension causes loss of consciousness and has often been observed together with vagally mediated bradycardia. Recurrent episodes of syncope often involve sympathetic nervous system dysfunction (Sheldon et al., 2015). While autoimmunity is typically not considered the cause of NCS in otherwise healthy individuals, when recurrent NCS occurs in the context of post-acute infectious syndromes, autoimmune disorders, or neurologic conditions, including autonomic neuropathy, autoimmune and immune-mediated etiologies should be considered.

Diagnosis is based primarily on clinical history, and a tilt table test can be utilized when the origin of syncope is unclear, although it can only point toward a susceptibility to vasovagal syncope and cannot definitively diagnose the condition (Sheldon et al., 2015). Similar to the treatment of POTS, the treatment of NCS involves increased fluid and salt intake, education about counterpressure maneuvers to be performed when prodromal symptoms occur, and wearing compression garments. For those with recurrent episodes with significant impact on daily functioning, medical management can include a trial of midodrine, fludrocortisone, beta-blockers, or selective serotonin reuptake inhibitors (SSRIs), while pacemaker implantation can be considered in treatment-refractory patients with severe and disabling NCS with a predominant cardioinhibitory component (Gampa and Upadhyay, 2018).

### 1.3 Orthostatic hypotension

Orthostatic hypotension (OH), defined as a reduction in blood pressure ≥20/10 mmHg that occurs within 3 min of standing or during a head tilt test, is often associated with symptoms commonly related to cerebral hypoperfusion, such as lightheadedness, dizziness, presyncope, or syncope (Freeman et al., 2011) (**Table 1**). OH can be associated with non-neurogenic causes (such as volume depletion or medication side effects) and neurogenic causes (such as senescence, neuropathic disorders, or neurodegenerative diseases). Medications, including vasodilators, nitrates, diuretics, phenothiazines, neuroleptics and antidepressants, can result in OH as a side effect (Medow et al., 2008). The severity of blood pressure reduction may also be influenced by the time of day, food ingestion, prolonged exposure to heat, fever, and alcohol consumption (Freeman et al., 2011). OH most often presents in the elderly, specifically one in five adults older than 60, and patients with neurodegenerative disorders (Freeman et al., 2011; Saedon et al., 2020). However, when OH occurs in the context of systemic autoimmune disorders, post-acute infectious syndromes, or neurologic disorders (such as autoimmune autonomic neuropathy or ganglionopathy), autoimmune and immune-mediated etiologies should be considered.

Mild cases of OH are commonly managed by discontinuing hypotensive medications and lifestyle changes, such as increasing water intake, avoiding alcohol, dietary changes, use of abdominal binders or leg stockings, and head-up tilt sleeping. The pharmacologic treatment approach for OH for patients with persistent symptoms is similar to that for patients with POTS and includes sympathomimetic agents (midodrine, yohimbine, vasopressin agonists, and clonidine), fludrocortisone, erythropoietin, pyridostigmine, selective serotonin reuptake inhibitors, and other medications (non-steroidal anti-inflammatory drugs (NSAIDs), antihistamines, caffeine, hydralazine, and ergotamine). Droxidopa, a norepinephrine precursor medication with combined central and peripheral alpha and beta agonist effects, was approved by the FDA for OH in 2014. It is indicated for the treatment of neurogenic OH and has shown improved symptoms and blood pressure elevation in four placebo-controlled randomized controlled trials (RCTs) (Brignole et al., 2018).

### 1.4 Inappropriate sinus tachycardia

Inappropriate sinus tachycardia (IST) is a chronic syndrome defined as an unexplained sinus heart rate of ≥100 bpm at rest or >90 bpm on average for 24 hours without orthostatic changes (Sheldon et al., 2015) (**Table 1**). IST may be associated with debilitating clinical symptoms, most often palpitations, and commonly occurs in women between the ages of 15 and 45. The pathophysiology of IST involves various proposed mechanisms, including an imbalance between sympathetic and parasympathetic inputs, accelerated intrinsic sinus node rate due to a deficient function of the acetylcholine and adenosine-sensitive potassium channels, and impaired baroreflex control (Ahmed et al., 2022). Since sinus tachycardia can be caused by various factors (including electrolyte abnormalities, dehydration, and hormonal abnormalities), these causes should be ruled out, and cardiac monitoring (such as an event monitor or an implantable loop recorder) should be used to correlate symptoms with heart rates (Ahmed et al., 2022). A 10-min stand test or a tilt table test can be used to distinguish IST from POTS, OH, and NCS (Olshansky and Sullivan, 2019), but sometimes, a patient may have more than one autonomic disorder, such as both POTS and IST.

The treatment of IST includes medications that reduce heart rate and symptoms, such as ivabradine (an I_f_ channel antagonist), beta-blockers, and calcium channel blockers. The combination of beta-blockers and ivabradine may be considered for ongoing management in some patients with IST (Olshansky and Sullivan, 2019). Sinus node modification, surgical ablation, and sympathetic denervation are not typically recommended as a part of routine care for patients with IST (Rodriguez-Manero et al., 2017).

### 1.5 Long COVID

Long COVID describes the health consequences of COVID-19 that persist beyond the initial infection. The World Health Organization defines post-COVID-19 conditions as symptoms that persist more than 12 weeks after probable or confirmed SARS-CoV-2 infection, which last at least 2 months and have no alternative explanations (Post COVID-19 condition (Long COVID), 2022). Similarly, the 2024 National Academies of Sciences, Engineering, and Medicine consensus defines Long COVID as “an infection-associated chronic condition that occurs after SARS-CoV-2 infection and is present for at least 3 months as a continuous, relapsing and remitting, or progressive disease state that affects one or more organ systems” (National Academies of Sciences, E. and Medicine, 2024; Ely et al.., 2024). Long COVID can follow either asymptomatic or symptomatic SARS-CoV-2 infection, and the current diagnosis is entirely clinical (National Academies of Sciences, E. and Medicine, 2024), given that there are no reliable and validated biomarkers available to clinicians at this time. A Long COVID Household Pulse Survey showed that the rate of Long COVID is nearly 7% of all adults—roughly 17 million people—as of March 2024 (Statistics, N.C.f.H, 2024). In another study in 2023, the National Health Interview Survey, 8.4% of adults in the USA reported ever having Long COVID, and 3.6% reported currently having Long COVID (Vahratian et al., 2024).

The pathophysiology of Long COVID is multifactorial but frequently involves autonomic dysfunction, including symptoms and signs such as palpitations, orthostatic intolerance, labile blood pressure, fatigue, headaches, and “brain fog” (Larsen et al., 2021). Consequently, many patients with Long COVID have POTS or OCADs (Blitshteyn and Whitelaw, 2021; Davenport et al., 2024), with nearly 70% of patients having a high autonomic symptom burden (Larsen et al., 2022). Autoimmune, inflammatory, and immune dysregulations are identified as other major pathophysiologic mechanisms of Long COVID, which, together with autonomic dysfunction, closely parallel the pathophysiology of POTS and OCADs. Increased prevalence of elevated serum autoimmune and inflammatory markers has been reported in patients with both POTS and Long COVID (El-Rhermoul et al., 2023), and neuroinflammation at the brainstem, specifically at the dorsolateral inferior medulla, has been suggested as a potential central nervous system localization for POTS and Long COVID (Blitshteyn, 2025). Moreover, consensus guidelines on the assessment and treatment of post-COVID autonomic dysfunction have been developed using non-pharmacologic and pharmacologic treatment options similar to POTS and OCADs unrelated to COVID-19 (Blitshteyn et al., 2022).

## 2 Autoimmunity

### 2.1 Autoimmune markers in POTS and other common autonomic disorders

The pathophysiology of POTS has been deemed largely heterogeneous and traditionally classified as neuropathic, hypovolemic, and hyperadrenergic (Low et al., 2009). In the past decade, however, investigators zeroed in on autoimmunity as one of the major mechanisms. Patients with POTS were found to have a higher prevalence of various non-specific autoimmune markers, including antinuclear antibodies and comorbid autoimmune disorders, than the general population (Blitshteyn, 2015). More specifically to the autonomic nervous system, ganglionic N-type and P/Q-type acetylcholine receptor antibodies, alpha 1, beta 1, and beta 2 adrenergic antibodies, muscarinic M2 and M4 antibodies, angiotensin II type 1 receptor antibodies, and opioid-like 1 receptor antibodies have been identified in patients with POTS and OCADs (Thieben et al., 2007; Li et al., 2014; Watari et al., 2018; Yu et al., 2018; Gunning et al., 2019; Kharraziha et al., 2020). Many of these antibodies have also been identified in patients with chronic fatigue syndrome, small fiber neuropathy, complex regional pain syndromes, and cardiovascular disorders—conditions that have overlapping clinical features with POTS.

### 2.2 Comorbidity with undifferentiated connective tissue disease

POTS and OCADs are commonly comorbid with other autoimmune disorders, with the most common being Hashimoto’s thyroiditis (Blitshteyn, 2015). Their association with Sjögren’s syndrome, antiphospholipid syndrome, and celiac disease has also been reported (Schofield et al., 2014; Penny et al., 2016; Mannan and Pain, 2023). In addition, many patients with autonomic dysfunction, small fiber neuropathy, and positive autoimmune or inflammatory markers are diagnosed with undifferentiated connective tissue disease (UCTD) when they do not meet the diagnostic criteria of defined autoimmune disorders, such as systemic lupus erythematosus, mixed connective tissue disease, Sjögren’s syndrome, systemic sclerosis, polymyositis, dermatomyositis, or rheumatoid arthritis. In clinical practice, the presence of undifferentiated connective tissue disease can be common.

Like POTS, UCTD predominantly affects women of reproductive age and is thought to be heterogeneous in mechanisms and presentations. UCTD is caused by an autoimmune etiology and may precede the onset of lupus or another defined classical autoimmune disease. UCTD includes the following diagnostic criteria: 1) clinical presentation suggestive of a defined connective tissue disease, but not meeting its criteria; 2) positive serological markers on two separate occasions, including positive antinuclear antibody marker; and 3) the duration of symptoms is at least 3 years (Mosca et al., 1999).

Positive serological markers are essential in the diagnostic criteria for UCTD and should include routine screening tests, such as complete blood count, C-reactive protein (CRP), erythrocyte sedimentation rate (ESR), serum creatinine, urinalysis with microscopic analysis, rheumatoid factor (RF), antinuclear antibodies (ANAs), anti-Ro/SSA/anti-SSB antibodies, and anti-U1-RNP (Marwa and Anjum, 2025). Treatment typically includes symptomatic management with non-steroidal anti-inflammatory medications, such as ibuprofen, naproxen, and celecoxib; corticosteroids, such as prednisone, methylprednisolone, and hydrocortisone; calcium channel blockers, such as diltiazem and nifedipine; and immunomodulatory therapy with an anti-malarial drug, hydroxychloroquine. In more severe cases, immunosuppressive medications, such as methotrexate and azathioprine, can be used, especially when there is evidence of significant organ damage or involvement (Rubio and Kyttaris, 2023). Further research is needed to elucidate whether POTS and OCADs with positive autoimmune markers represent a sizable subset of patients with UCTD, what longitudinal monitoring is required in this subset, and whether early intervention with treatment (such as hydroxychloroquine or low-dose naltrexone) can alter the natural history and potentially prevent further progression of the disease process.

### 2.3 Association with autonomic neuropathy

POTS and OCADs can often occur as part of, or in the context of, autonomic neuropathy. Experts who originally described POTS have considered it to be a limited or restricted form of autonomic neuropathy (Schondorf and Low, 1993; Vernino et al., 2008). Approximately half of patients with POTS have a length-dependent distribution (Low et al., 1994; Low et al., 2009) with distal postganglionic sudomotor denervation demonstrated by the quantitative sudomotor axon reflex test (QSART) or the thermoregulatory sweat test (Low, 1993). These tests commonly reveal sudomotor denervation in the feet and toes: adrenergic impairment in the lower extremity can be seen in neuropathic POTS as impaired norepinephrine spillover in the leg, while the arm response remains normal (Jacob et al., 2000). However, a non-length-dependent or patchy distribution of small fiber neuropathy can also occur, especially in conjunction with systemic autoimmune disorders (Gemignani et al., 2022). Autoimmune and immune-mediated etiologies have been suggested as among the major underlying mechanisms in autonomic neuropathy, with immunotherapy being recommended as the first-line treatment (Gavrilova et al., 2022; Maier et al., 2022; Gendre, 2024; Nakane et al., 2024).

### 2.4 Autoimmunity in Long COVID

Autoimmunity has been implicated as one of the major mechanisms of Long COVID, leading to a higher risk, overall incidence, and range of autoimmune conditions after SARS-CoV-2 infection (Sharma and Bayry, 2023). A variety of antibodies have been linked to Long COVID, including autoantibodies to inflammatory cytokines such as IgG to IL-2, D8B, thyroglobulin, and IFNδ (Rojas et al., 2022; El-Rhermoul et al., 2023; Peluso and Deeks, 2024). These autoantibodies have been associated with anti-SARS-CoV-2 IgG antibodies (Rojas et al., 2022; El-Rhermoul et al., 2023; Peluso and Deeks, 2024). G protein-coupled receptor antibodies, including against alpha- and beta-adrenergic antibodies and muscarinic antibodies, previously identified in patients with POTS, as well as autoantibodies to antinuclear and extractable nuclear antigens, have also been found in patients with Long COVID (Wallukat et al., 2021; El-Rhermoul et al., 2023; Son et al., 2023). The pro-inflammatory mediators, non-specific antibodies, and antibodies important to the function of the autonomic nervous system are thought to be implicated in the development of post-COVID autonomic disorders, such as POTS and OCADs (El-Rhermoul et al., 2023).

## 3 Immunotherapies

### 3.1 Immunologic therapies and ongoing clinical trials for POTS and other common autonomic disorders

#### 3.1.1 Immunoglobulin

Intravenous immunoglobulin (IVIG) or subcutaneous immunoglobulin (SCIG) comes from a concentrate of pooled immunoglobulins derived from 1,000 to 100,000 healthy donors and serves as an immunomodulating therapy that can neutralize autoantibodies, reduce cellular immunity, and decrease endothelial inflammation by increasing IgG levels in the bloodstream (Danieli et al., 2025). Immunoglobulins play a vital role in humoral adaptive immunity, and therefore, IVIG reflects a collective exposure of the donor population to their environment and can be expected to contain various antibodies of multiple specificities against a broad spectrum of infectious agents (bacterial, viral, and others), self-antigens, and anti-idiotype antibodies. The composition of IVIG products closely corresponds to that of immunoglobulins in normal human plasma, especially IgG (along with its subclasses), IgA, traces of other Igs, cytokines, and soluble receptors (Perez et al., 2017).

IVIG has been indicated as a replacement therapy in immunodeficiencies, as an immunomodulatory and anti-inflammatory therapy for immunomodulation in hematological and organ-specific autoimmune disorders, and as an anti-inflammatory in rheumatic inflammatory conditions and infectious neurologic disorders. It has also been utilized as a hyperimmune therapy against specific infectious agents (Perez et al., 2017).

Given its widespread use in neurologic conditions [such as Guillain–Barré syndrome, chronic inflammatory demyelinating polyneuropathy (CIDP), acute disseminated encephalomyelitis (ADEM), multifocal motor neuropathy (MMN), dermatomyositis, and myasthenia gravis], IVIG has also been used successfully in treating less common peripheral neuropathies, such as autoimmune autonomic ganglionopathy (AAG) and autoimmune autonomic neuropathy (AAN) (Gibbons et al., 2008; Dalakas, 2021). To this end, a trial of IVIG or SCIG seems reasonable in POTS—a restricted form of AAN—and OCADs, especially in patients with comorbid small fiber neuropathy (SFN), UCTD, or systemic autoimmune disorder.

Over the past decade, case reports and case series describing the benefits of IVIG in POTS and OCADs have been accumulating. All reported reduced autonomic symptoms, orthostatic intolerance, fatigue, functional impairment, and lowered antibody titers when available. Similar findings were observed in other case reports of IVIG or SCIG in patients with OCADs (**Table 2**). Importantly, these reports suggest that IVIG and SCIG are well-tolerated without significant serious adverse events, although side effects, including post-infusion headache and flu-like symptoms, were common. Slower infusion rates with pretreatment with IV saline, antihistamines, and anti-inflammatories may mitigate these side effects and improve tolerability (Guo et al., 2018).

**Table 2:** Immunotherapy in POTS, OCADs, and Long COVID: review of literature.

Indication	Study design	Immunotherapy, administration, dosage, and course	Outcome measures	Key findings	Adverse effects
POTS	Double-blind randomized controlled trial of IVIG (n = 16) vs. albumin (n = 14) (Vernino et al., 2024)	IVIG (Gamunex-C^®^) 0.4 g/kg for 12 weeks. 1. Weekly for 4 weeks. 2. q2 weeks for 8 weeks.	- Change in Symptoms Measured by Change in COMPASS-31 Score from baseline to week 13. - Orthostatic vitals (active stand test) and laboratory studies for safety were collected at screening, baseline, and weeks 5, 13, and 15.	- No difference between treatment groups at week 13 in scores. - IVIG group had a non-statistically significant higher response rate (46.7% vs. 38.5%) vs. placebo.	- No difference in AE between patients vs. controls - Mild headache - One patient with pneumonia
	Case report (Goto et al., 2021) n = 1	1. IVIG 400 mg/kg/day for 5 days. 2. IV 0.5 g/kg initiated after 1 month, every q5–6 weeks.	- Change in serum antibody testing. - Change in vital signs on HUT test, at baseline and post-treatment. - Change in ability to do daily activities of living.	- Decrease in anti-gAChR antibody index at baseline from 2.162 to 1.438. - Patient’s HUT showed HR change from lying and standing, which reduced from 56 to 34 bpm.	None reported
	Case series (Rodriguez et al., 2021) n = 6	IVIG 0.4 g/kg 1. Daily for 5 days (2 g/kg maximum dose). 2. Given over 2 days monthly (0.8 g/kg maximum dose).	- Change in heart rate increase (bpm) after 10 min of HUT test, duration (min) of TST, and anhidrotic area (%) in the TST at baseline and 6 months after IVIG treatment. - Change in standardized symptom questionnaires from baseline to 6 months of IVIG treatment.	- Symptom severity was reduced by nearly 40%. 83.3% had improved performance, exercise tolerance, and, later on, gastrointestinal symptoms. - Autonomic function testing showed improved cardiovascular functioning by 50% and a reduction of anhidrotic areas by one-third.	- Aseptic meningitis and hospitalization (n = 2) - Hypertension (n = 2)
	Case report (Pitarokoili et al., 2021) n = 1	1. IVIG 2 g/kg for 5 days. 2. IV 1 g/kg given 11 times, at a rate of 2–3 g/h. 3. Subcutaneous 0.25 g/kg, changed to weekly for 6 months.	- Change in HUT test. - Change in COMPASS-31 questionnaire. - Change in antibody titers.	- Reduction of serum antibodies. - Improvement COMPASS-31 scores. - Cessation of syncopal episodes while standing.	No major AE
	Case report (Wells et al., 2020) n = 1	PLEX (3 L of plasma with 4% albumin) given over 2–4 hours for 6 sessions within a 2-week period.	- Change in COMPASS-31 questionnaire. - Change in OHSA and OHDAS scores. - Change in CANTAB score. - Change in 10-min tilt table test.	- Improvement in COMPASS-31 (40%), OHSA (38%), and OHDAS (29%) scores. - CANTAB score indicated some improvement in attention, alertness, and memory metrics. - Tilt table test only showed minor improvements when reassessed post-2 weeks of treatment. - Symptoms returned within 1 month of PLEX treatment, and pt was restarted on a maintenance dose every q2–3 weeks over 18 months.	None
	Case series (Kesterson et al., 2023) n = 7	SCIG (5/7) PLEX q2 weeks or monthly for at least 3 months.	- Change in COMPASS-31 score and FAS score from baseline to 3–12 months post-treatment.	- Average 50% reduction in COMPASS-31 score, 217% increase in FAS scores within 3 to 9 months of treatment. - 6 pts reduced or discontinued oral medications for POTS. - 5 pts had a FAS score higher than 80% and able to return to work or school.	No major AE
	Case report (Weinstock et al., 2018) n = 1	Immunoglobulin (Privigen^®^) IV 1.5 g/kg monthly for 1 year.	- Change in 10-point Likert scale to score severity and frequency of symptoms.	Improved syncope, body pain, weakness, vertigo, syncope, GI symptoms, and tinnitus. - After 10 IVIG infusions, resolution of tachycardia on HUT and improvement in sudomotor function.	No major AE
	Case report (Hendrix et al., 2021) n = 1 POTS with seronegative ankylosing spondylitis	Adalimumab SC, unknown dose and duration.	- Change in Likert scale, to score severity and frequency of symptoms, from baseline and after treatment.	- Complete resolution of POTS symptoms within days to 1 week of treatment initiation.	None
	Case report (Zadourian et al., 2018) n = 1	1. Rituximab IV 375 mg/m^2^ q4 weeks for 1 year. 2. PLEX 2–3× per week for 1 year.	Not specified.	- Improvement in symptoms, such as going from being bedbound to walking 2 miles, exercising daily for 1 hour, and returning to work.	None
OCADs	Open-label cohort study (Pasricha et al., 2024) in AD n = 32	Immunoglobulin IV 2 g/kg monthly for at least 3 months.	- Change in upper gastrointestinal symptom severity and QoL every 2 months for 2 years.	- Improvement of OTE scores, with a mean of 1.8 (SD 3.2), was significantly better than 0 at baseline (p = 0.004). - The PAGI-QOL indicated “great or very great deal better” (p < 0.001) and a clinically significant response (p = 0.001).	Greater than 60% reported side effects; none were life-threatening.
	Case series (Flanagan et al., 2014) in autoimmune GI dysmotility n = 23	Immunoglobulin IV 0.4 g/kg given over 3 days or methylprednisolone IV 1 mg daily for 3 days, then weekly or both for 6–12 weeks.	- Response was defined subjectively (symptomatic improvement) and objectively (gastrointestinal scintigraphy/manometry studies).	- 74% had improved symptoms and scintigraphy, five; symptomatic alone, eight; scintigraphy alone, four. - 6/7 with repeat autonomic testing after treatment demonstrated improvements.	Aseptic meningitis (n = 1)
	Case series (Schofield and Chemali, 2019) in AD n = 38	Immunoglobulin IV 0.25 g/kg weekly for at least 3 months, then increased to 1 g/kg/month.	- Change in disease activity, measured by COMPASS-31 and FAS scores, from baseline and regular intervals. - Repeat skin biopsies after 12 months or more of IVIG therapy.	- Improved in FAS and COMPASS-31 scores reported in 83.5% of patients. - Pretreatment average FAS score changed from 21% (mostly bedridden) to 74% (able to return to work or school) in 1 year. - Improved sweat gland and/or epidermal nerve fiber density in 2 out of 4 patients 1 year after IVIG.	- Headache - Neck pain - Fatigue - Myalgias - Aseptic meningitis - Transaminitis - MCAS flare
	Case report (Sokmen et al., 2023) in AN with Sjögren’s syndrome n = 1	Immunoglobulin 1. IV 2 g/kg given over 5 days, then 0.4 g/kg/month × 1.5 years.	- Change in disease activity, measured by COMPASS-31 score and FAS score, from baseline.	- After 6 months, patient could walk long distances; COMPASS-31 improved from 51 to 11 after 1.5 years on IVIG.	None
	Case report (Kataria et al., 2023) in autonomic dysfunction in Sjögren’s syndrome n = 1	Oral steroid with dose and course not specified.	Not specified.	- Patient reported significant clinical improvement after midodrine, and Florinef failed to improve autonomic symptoms.	None
	Case series (Pang et al., 2017) in acute AN n = 10	Immunoglobulin IV 2 g/kg given for 5 days. With or without IV methylprednisolone or dexamethasone.	Change in autonomic nerve function tests and modified Rankin scale.	- Sensory and motor symptoms recovered significantly, and autonomic symptoms were reduced. - 9 patients improved after treatment of IVIG and IV steroids. - 4 patients with severe illness worsened.	None
	Case series (Goodman, 2019) in autonomic dysfunction in Sjögren’s syndrome n = 4	Immunoglobulin IV 0.4 to 0.8 g/kg monthly; rituximab IV 1 g on days 1 and 15.	- Change in autonomic function testing and CASS score.	- Marked improvement in clinical and functional status correlated with improved autonomic testing in all patients.	None
	Case series (Oishi et al., 2021) in autonomic dysfunction in neurosarcoidosis n = 11	Oral prednisolone with or without IVIG or IV methylprednisolone.	Not specified.	- 10/11 of patients were categorized as responsive to immunotherapy by the authors.	None
	Case report (Goodman, 2014) in AN n = 1	IV Methylprednisolone for 5 days followed by IV immunoglobulin × 5 days.	- Change in autonomic testing and symptomatology.	- Substantial improvement in symptoms. - Post-treatment autonomic testing improved.	None
	Case report (Bouxin et al., 2019) in autoimmune AN n = 1	IVIG 2 g/kg/day, then TPE every other day for 6 sessions; then rituximab 1,000 mg twice, 2 weeks apart; then prednisone 60 mg daily	- Change in COMPASS-31 score.	- Improved symptoms and COMPASS-31 score after treatment with each medication sequentially.	None
	Case series (Tiongson et al., 2016) in autoimmune AN n = 2	IVIG 2 g/kg monthly Rituximab IV 750 mg/m^2^ twice, 2 weeks apart.	- Change in autonomic function tests, EMG, and symptoms.	- Improved symptoms after IVIG and Rituxan; improved functional status and neurologic exam.	Abdominal cramps
Long COVID	Case report (Novak, 2020) n = 1	IVIG 2 g/kg monthly; then after 2 months, 1 g/kg/month.	- Change in symptomatology.	- Resolution of some symptoms. - Headaches/fatigue improved by 50%.	Headache
	Placebo case control study for IVIG (n = 9) vs. placebo (n = 7) (McAlpine et al., 2024)	Immunoglobulin IV 2 g/kg q3 weeks for 10 months.	- Change in autonomic symptoms, skin biopsy, iCPET testing, and labs.	- Resolution (6/9) or improvement (3/9) in clinical response (p = 0.001) and significant clinical response in neuropathic symptoms (9/9) with IVIG compared to no IVIG (3/7; p = 0.02).	None
	Prospective cohort study (Stein et al., 2025) n = 20	Immunoadsorption Five sessions (4.5–9 hours each) given over 10 days, with no more than 2 days apart.	- Change COMPASS-31, QoL, and FFS scores. - Change in muscle fatigue and vascular dysfunction, assessed by hand grip strength (HGS) on dynamometer and EndoPAT^®^ measurements.	- Improvement in SF-36 scores between 2 and 3 months, with significant improvement found over 6 months. - 70% of participants were responders at 4 weeks post-treatment. - Improved autonomic symptoms (p = 0.001); increased HGS 6 months post-treatment.	Internal jugular vein thrombosis (n = 1)
	Case series (Tomisti et al., 2023) n = 2	Convalescent plasma (CP) IV 300 mL, 3 doses over 15 days: 1. 3,332.6 BAU/mL 2. 1,794.2 BAU/mL 3. >5,680 BAU/mL	- Cycle threshold (CT) values from PCR NPS. - Symptomatology. - Chest CT scan.	- Negative NPS 5 days after last dose of C - Complete resolution of symptoms 1 month after C	None
		Convalescent plasma (CP) IV 500 mL, 2 doses given 5 days apart. 1. 5,680 BAU/mL 2. 4,556 BAU/mL	- Cycle threshold (CT) values from PCR NPS. - Symptomatology. - Chest CT scan.	- Complete resolution of fever with clinical improvement 1 day after the first dose of C - Negative NPS 2 days after last dose of C	None
	Case report (Seeley et al., 2025) n = 1	TPE daily for 5 days.	- Change in cognitive function, measured by MoCA and CANTAB. - Change in ambulation distance (m).	- Pain, walking, and cognitive function, assessed by MoCA and CANTAB, improved.	None
	Placebo-blinded randomized clinical trial (A phase 2 randomized, double-blinded, placebo-controlled study to evaluate the efficacy and safety of efgartigimod IV in adult patients with post-COVID-19 postural orthostatic tachycardia syndrome (POTS, 2022; SE, 2024) n = 53	Efgartigimod IV 10 mg/kg weekly for 24 weeks.	- Change in COMPASS-31 and MaPS. - Change in laboratory test results and vital sign measurements. - Change in fatigue, cognitive function, etc.	- No clinically meaningful improvement when compared to placebo for the MaPS score and COMPASS-31. - Clinical trial was closed prematurely, and further outcome measures are yet to be released.	Unknown
	Open-label prospective study (O’Kelly et al., 2022) n = 38	LDN 1–3 mg po daily for 2–3 months.	- Change in Likert scale: sleep, concentration, pain/discomfort, mood, energy levels, limitation in activities of daily living, and perception of overall recovery from COVID.	- Significant reduction in reported low pain, mood, chest tightness, and cough (p < 0.05).	- Diarrhea - Fatigue - 2 patients discontinued it due to AE
	Observational open-label prospective study (Isman et al., 2024) n = 36	LDN 4.5 mg po QHS daily for 12 weeks.	- Reduction of fatigue measured by Chalder fatigue scale and SF-36 at 12 weeks post-treatment.	- Significant increase in SF-36 survey scores after 12 weeks of treatment (p < 0.0001); significant decrease in Chalder fatigue scale scores after 12 weeks of treatment (p < 0.0001). - 52% were responders at 12 weeks.	- Nausea - Fatigue - Dizziness - Insomnia - Diarrhea - SOB

POTS, postural tachycardia orthostatic syndrome; OCADs, other common autonomic disorders; AD, autoimmune dysautonomia; AN, autonomic neuropathy; AE, adverse event; SAE, serious adverse event; LDN, low-dose naltrexone; COMPASS-31, Composite Autonomic Symptom Score 31; FAS, functional ability scale; CASS, Composite Autonomic Severity Score; HUT, head-up tilt; TST, thermoregulatory sweat test; ECG, electrocardiography; NPS, nasopharyngeal swab; MoCA, Montreal Cognitive Assessment; CANTAB, Cambridge Neuropsychological Test Automated Battery; MaPS, Malmo POTS Symptom Score; PROMIS, Patient-Reported Outcomes Measurement Information System; SF-36, 36-Item Short Form Health Survey; PAGI-QoL, Patient Assessment of Upper Gastrointestinal Disorders—Quality of Life; OTE, overall treatment effectiveness; SOB, shortness of breath; SQ, subcutaneous; HGS, hand grip strength; OHSA, Orthostatic Hypotension Symptom Assessment; OHDAS, Orthostatic Hypotension Daily Activity Scale.

Recently, a small randomized controlled study found no significant benefit of 16 patients treated with IVIG vs. 14 patients treated with albumin with autoimmune POTS despite a trend toward a higher response rate in the IVIG-treated group (Vernino et al., 2024). However, the true benefit of IVIG may not have been captured, as the study was underpowered, used lower IVIG doses than those for autoimmune disorders, was of short duration, and had other major limitations (Chemali et al., 2024). Further research with large, multicenter, randomized controlled trials of longer duration and addressing major limitations is needed to provide a comprehensive and objective assessment of the efficacy of IVIG in patients with POTS (Chemali et al., 2024).

#### 3.1.2 Plasma exchange

Therapeutic plasma exchange (TPE), also known as plasmapheresis, is a technique that rapidly removes circulating autoantibodies and other humoral factors from the vascular compartment and has been used as the first effective acute treatment for neurologic disorders, such as Guillain–Barré syndrome and myasthenia gravis, before intravenous immunoglobulin became available (Osman et al., 2020). It is still used when IVIG is not available or ineffective in a variety of neuroimmune disorders, including CIDP and autoimmune encephalitis (Osman et al., 2020). Isolated cases of a total of five patients with severe POTS have been described in scientific literature; their POTS symptoms improved significantly with TPE, with patients being able to return to work and other daily activities, such as walking and exercising (Zadourian et al., 2018; Wells et al., 2020; Kesterson et al., 2023) (**Table 2**). Despite no significant adverse events reported, further studies are necessary to determine the efficacy and safety of TPE in patients with severe POTS and OCADs.

#### 3.1.3 Biologic immunotherapies

Biologic therapies in POTS and OCAD cases have not been explored in-depth but may be a good option to explore in patients with severe symptoms. Rituximab, an anti-CD20 monoclonal antibody, could be of benefit in autoimmune autonomic disorders, as it targets B cells that are created by the adaptive immune system and are responsible for autoantibody production. There are limited data on its use in POTS and OCADs; however, it has been utilized in select cases with other autoimmune neurologic conditions with autonomic involvement (Hollenbeck et al., 2011; Bouxin et al., 2019). Currently, rituximab use has been reported in one POTS patient and three OCAD patients (Tiongson et al., 2016; Zadourian et al., 2018; Goodman, 2019). All patients reported autonomic symptomatic resolution, with two demonstrating absence or a decrease in autoimmune antibodies post-treatment.

Adalimumab is a monoclonal antibody against tumor necrosis factor-alpha (TNF-α), a pro-inflammatory cytokine made by the innate immune system, that is responsible for regulating inflammation, cell differentiation, and tissue destruction. It is approved by the FDA for the treatment of rheumatoid arthritis, inflammatory bowel disease, and other autoimmune and inflammatory disorders. One case report described the use of adalimumab in a patient with POTS and seronegative ankylosing spondylitis, which led to complete symptom resolution of POTS symptoms within 1 week of the induction dose and no adverse effects (Hendrix et al., 2021) (**Table 2**).

Tocilizumab is an IL-6 receptor antagonist that activates the JAK/STAT3 pathway and regulates inflammation, B-cell activation, and autoantibody production. Although it has been used in neurologic and autoimmune disorders, such as neuromyelitis optica spectrum disorder (Du et al., 2021) and rheumatoid arthritis (Syngle et al., 2015), it has yet to be explored in POTS and OCADs. Currently, the application of biologic therapies in POTS and OCADs remains extremely limited, primarily due to the inaccessibility of these agents, high cost, and potential for adverse effects, but future pharmaceutical research and investment in clinical trials are warranted to assess their full therapeutic potential. Notably, there is one phase II double-blind placebo-controlled clinical trial investigating a novel monoclonal antibody against natriuretic peptide receptor 1 that began recruiting POTS patients in late 2024 (Patients with postural orthostatic tachycardia syndrome, 2024) (**Table 3**).

**Table 3:** Ongoing and pending immunotherapy trials for POTS and Long COVID.

Identifier	Location	Indication	Immunotherapy	Administration, dosage, and course	Selective outcome measures
NCT06593600 (Patients with postural orthostatic tachycardia syndrome, 2024)	Europe	POTS	NPR1 antagonist monoclonal antibody	Single high- or low-dose SQ injection	- HR change from supine to standing (DeltaHR) at days 8, 15, and 29. - Serum concentration over 90 days. - AE occurrence and severity over 90 days.
NCT06305793 (RECOVER-AUTONOMIC (IVIG): randomized trial of the effect of IVIG versus placebo on long COVID symptoms, 2024)	Durham, NC, USA	Post-COVID autonomic dysfunction NIH-RECOVER	Immunoglobulin (Gamunex^®^)	IV 2 g/kg monthly for 9 months (36 weeks)	- Change in OHQ/OIQ, COMPASS-31, MaPS, PROMIS-29, VOSS, PASC Symptom Questionnaire from baseline to end of treatment. - Change in Active Stand Test (BP and HR) and 6-min walk test. - Incidence of SAEs and ESIs up to 3 months post-treatment. - Changes in autonomic function testing from baseline to end of treatment.
NCT06524739 (Double-blind, randomized, placebo-controlled phase 3 study evaluating efficacy and safety of igPro20 (Subcutaneous immunoglobulin, HIZENTRA®) in post-COVID-19 postural orthostatic tachycardia syndrome (POTS), 2024)	Multiple sites in USA and Canada	Post-COVID POTS	Immunoglobulin (HIZENTRA^®^)	SCIG IgPro20, a 20% ready-to-use liquid formulation	- Proportion of participants no longer meeting diagnostic criteria of post-COVID POTS as measured by standardized standing test at baseline vs. week 25. - Change of COMPASS-31 score at week 25. - Number and percentage of participants with TEAEs for up to 57 weeks post-treatment.
NCT05841498 (A single-blinded sham-controlled crossover trial to evaluate the effect of immunoadsorption on post-corona virus disease (COVID)-syndrome, 2023)	Mainz, Germany	Long COVID-19	Immunoadsorption	5 sessions of central venous catheter	- Improvement of post-COVID symptoms, fatigue, and cognitive impairment as measured by various questionnaires at 2 weeks post-IA. - Change of HGS measured as hand grip strength test with a dynamometer at 2 weeks post-IA. - Number of SAEs and discontinuations at 2 weeks post-IA. - Prevalence of anti-adrenergic and anti-muscarinic autoantibodies at baseline; concentration of autoantibodies pre- and post-IA treatment.
NCT05710770 (Double-blinded, randomized, sham-controlled trial of immunoadsorption (IA) in patients with chronic fatigue syndrome (CFS) including patients with post-acute COVID-19 CFS (PACS-CFS), 2023)	Berlin, Germany	Post-COVID CFS	Immunoadsorption	5 sessions over 9–12 days	- Improvement in physical and mental fatigue as measured by the Chalder fatigue score scale and other questionnaires at 3 months post-IA. - Number of TEAEs, SAEs, and discontinuations at 1, 3, and 6 months post-IA. - Improvement in COMPASS-31 scores at 10 days and 3 and 6 months post-IA. - Improvement in autonomic dysfunction by measuring the Schellong Test at 3 and 6 months post-IA. - Changes in serum autoimmune/inflammatory biomarkers at 3 and 6 months post-IA.
NCT05220280 (Long-term follow-up of a randomized multicenter trial on impact of imatinib and infliximab on long-COVID in hospitalized COVID-19 patients, 2022)	Finland	Hospitalized COVID-19 patients	Infliximab vs. imatinib	Infliximab IV 5 mg/kg × 1 dose Imatinib: 400 mg po qd × 14 days	- Symptom questionnaire at 1 and 2 years of follow-ups. - EQ-5D-5L questionnaire at 1 and 2 years of follow-ups. - Lung function by spirometry and diffusing capacity. - 6MWT. - Whole-genome genotyping.
ISRCTN46454974 (A research trial to find out if tocilizumab helps adults with Long Covid feel better, 2025)	United Kingdom	Long COVID-19	Tocilizumab	SQ q weekly or fortnightly × 12 weeks	- Questionnaires to assess symptoms or physical and mental health, brain fog, and physical performance. - Breathing test and imaging.
NCT06631287 (Randomized double-blind placebo-controlled trial EValuating baricitinib on PERSistent NEurologic and cardiopulmonary symptoms of long COVID (REVERSE-LC, 2024)	Nashville, TN, USA	Long COVID-19	Baricitinib (OLUMIANT^®^)	4 mg PO daily for 24 weeks	- CNS-Vital Signs Global Cognitive Index at 6 months. - Exercise capacity, including the 6MWT at 6 and 12 months. - CPET at 6 and 12 months. - QoL and other symptom measures at 6 and 12 months. - Orthostatic intolerance using the OIQ at 3, 6, and 12 months. - COMPASS-31 scores at 3, 6, and 12 months.
NCT05877508 (Aerium, 2023)	San Francisco, CA, USA	Long COVID-19	Anti-SARS-CoV-2 monoclonal antibodies	IV 1,200 mg since dose	- Change in symptom scores via various questionnaires. - Change in COMPASS-3 Score from baseline to day 90. - Change in 6MWT and active stand test from baseline to day 90. - Change in CRP, ESR, D-dimer, and fibrinogen from baseline to day 90.

POTS, postural orthostatic tachycardia syndrome; OCHOS, orthostatic hypoperfusion syndrome; SFN, small fiber neuropathy; ME, myalgic encephalomyelitis; CFS, chronic fatigue syndrome; PASC, post-acute sequelae of SARS-CoV-2 infection; HR, heart rate; ADA, antidrug antibody; AE, adverse event; OHQ, Orthostatic Hypotension Questionnaire; OIQ, Orthostatic Intolerance Questionnaire; COMPASS-31, Composite Autonomic Symptom Score 31; MaPS, Malmo POTS Symptom Score; BP, blood pressure; PROMIS, Patient-Reported Outcomes Measurement Information System; SAE, severe adverse event; VOSS, Vanderbilt Orthostatic Symptom Score; TEAE, treatment-emergent adverse event; ECG, electrocardiogram; MoCA, Montreal Cognitive Assessment; QoL, quality of life; EQ-5D-5L, EuroQoL 5-level EQ-5D version; 6MWT, 6-min walk test; CPET, cardiopulmonary exercise testing; CRP, C-reactive protein; ESR, erythrocyte sedimentation rate; HGS, hand grip strength.

#### 3.1.4 Other traditional immunomodulators

Although immunomodulating therapies have not been typically included in the standard pharmacologic approaches for POTS and OCADs, these treatment options have been gaining utility, especially in the context of comorbid UCTD, systemic autoimmune disorders, and Long COVID. These pharmacotherapies include oral, IV, and subcutaneous (SQ) corticosteroids, low-dose naltrexone, and immunosuppressants, such as hydroxychloroquine. These medications may be attractive, as they have more established safety profiles, clinical familiarity, and easier accessibility through insurance coverage compared to other immunologic therapies. Corticosteroids are effective in reducing inflammation and autoimmunity and have been used for decades for acute exacerbation of multiple sclerosis, neuromyelitis optica, myasthenia gravis, and others. They have been reported for treatment of autonomic dysfunction either as monotherapy or in combination with other immunotherapies in patients with neurologic Sjögren’s syndrome and autonomic neuropathy associated with neurosarcoidosis (Flanagan et al., 2014; Goodman, 2014; Pang et al., 2017; Oishi et al., 2021; Kataria et al., 2023). Improvement with corticosteroids has been observed in these small case series; however, long-term use is not recommended due to significant steroid-induced side effects, including long-term risk of diabetes, osteoporosis, hypertension, and Cushing’s syndrome (Buchman, 2001).

Naltrexone is a potent mu-opioid receptor antagonist at high doses, primarily used to prevent relapse in opioid use disorder. Below 5 mg, low-dose naltrexone (LDN) acts as a glial modulator, inhibits Toll-like-receptor-4 (TLR-4), and only partly antagonizes opioid receptors. Its anti-TLR-4 effects inhibit proinflammatory cytokine production, while its partial opioid receptor downregulation signals for increased opioid production and can downregulate the immune system in POTS and OCADs (Li et al., 2018; Trofimovitch and Baumrucker, 2019). There are no clinical trials on the use of LDN in POTS and OCADs, with only one case report documenting beneficial LDN use in POTS (Weinstock et al., 2018) (**Table 2**). Clinical experience suggests that many patients report improvement in chronic pain, chronic fatigue, and mast cell-related symptoms with the use of LDN.

Antimetabolite immunosuppressants, such as mycophenolate mofetil, azathioprine, or Hydroxychloroquine, could also be of potential therapeutic benefit in autoimmune POTS and OCADs, but the use of these medications in patients with POTS and OCADs has not been investigated. Anecdotal reports of patients with POTS and OCADs and comorbid autoimmune disorders, such as UCTD and Sjögren’s syndrome, suggest that there may be potential benefits in this subset of patients.

### 3.2 Immunologic therapies and ongoing clinical trials for Long COVID

Immunotherapies documented in Long COVID case reports and cohort studies include IVIG, immunoadsorption, convalescent plasma (CP), TPE, and LDN. Due to their proposed therapeutic role in autoimmune POTS and OCADs, these therapies could be considered potential therapeutic options for Long COVID-associated dysautonomia, but their use is extremely limited due to a lack of access and insurance coverage.

Three case reports have documented the utility of IVIG, TPE, and CP treatments in Long COVID. Novak reported improvement in headache and fatigue, with complete symptom resolution of all other symptoms (Novak, 2020). Minor adverse effects, such as headaches, were alleviated by dose down-titration. Tomisti et al. treated two patients with CP who reported complete symptom resolution within 1 month after their final treatment dose and reported no side effects (Tomisti et al., 2023). Lastly, Seeley et al. treated one patient with TPE who reported improved cognitive function, peripheral pain, and ambulation capacity from 5 to 12 m (Seeley et al., 2025). They also did not report side effects (**Table 2**).

Four prospective studies, although limited in sample size, have demonstrated clinical improvements in Long COVID and post-COVID syndromes following treatment with LDN (n = 38), immunoadsorption (n = 20), and immunoglobulin (n = 9) (O’Kelly et al., 2022; McAlpine et al., 2024; Stein et al., 2025). O’Kelly et al. conducted an open-label prospective study with 38 patients receiving 1 mg of LDN, assessing improved outcomes by self-reported questionnaires (O’Kelly et al., 2022). They found the biggest effect of symptom reduction in joint pain. Additionally, Isman et al. investigated LDN in an open-label prospective study with 36 Long COVID subjects over 12 weeks. They reported significant improvements in the patient’s quality of life and fatigue, measured by their 36-Item Short Form Health Survey (SF-36) and CFS scores. Approximately half of their participants were identified as clinical responders (Isman et al., 2024) (**Table 2**).

A placebo-controlled clinical trial was conducted for efgartigimod in 53 patients with post-COVID POTS, but preliminary outcomes showed no benefit of efgartigimod compared to placebo (A phase 2 randomized, double-blinded, placebo-controlled study to evaluate the efficacy and safety of efgartigimod IV in adult patients with post-COVID-19 postural orthostatic tachycardia syndrome (POTS, 2022). The clinical trial was stopped in 2024, and its outcome data have yet to be released (SE, 2024) (**Table 2**). Currently, eight immunotherapy clinical trials are ongoing for Long COVID and post-COVID autonomic disorders. These clinical trials are investigating IVIG, immunoadsorption, infliximab compared to imatinib, tocilizumab, baricitinib, and an anti-SARS-CoV-2 monoclonal antibody therapy. Four clinical trials are being held in North America (the USA and Canada), including one as part of the NIH-RECOVER autonomic study, with the other trials taking place in Germany, Finland, and the United Kingdom (Long-term follow-up of a randomized multicenter trial on impact of imatinib and infliximab on long-COVID in hospitalized COVID-19 patients, 2022; Aerium, 2023; A single-blinded sham-controlled crossover trial to evaluate the effect of immunoadsorption on post-corona virus disease (COVID)-syndrome, 2023; Double-blinded, randomized, sham-controlled trial of immunoadsorption (IA) in patients with chronic fatigue syndrome (CFS) including patients with post-acute COVID-19 CFS (PACS-CFS), 2023; Double-blind, randomized, placebo-controlled phase 3 study evaluating efficacy and safety of igPro20 (Subcutaneous immunoglobulin, HIZENTRA®) in post-COVID-19 postural orthostatic tachycardia syndrome (POTS), 2024; Randomized double-blind placebo-controlled trial EValuating baricitinib on PERSistent NEurologic and cardiopulmonary symptoms of long COVID (REVERSE-LC, 2024; RECOVER-AUTONOMIC (IVIG): randomized trial of the effect of IVIG versus placebo on long COVID symptoms, 2024) (**Table 3**).

### 3.3 Immunologic therapies for ME/CFS

ME/CFS has overlapping clinical features with POTS, OCADs, and Long COVID and is therefore relevant to this review. A number of immunologic therapies have been studied in ME/CFS, including IVIG, SCIG, and IgG depletion by immunoadsorption (McAlpine et al., 2024; Sjogren et al., 2024; Stein et al., 2025). Four double-blind placebo-controlled RCTs of IVIG for ME/CFS were conducted in the 1990s: one study reported that immunoglobulin is effective in a “significant number of patients”, and another reported that IVIG “is unlikely to be of clinical benefit in CFS” (Lloyd et al., 1990; Peterson et al., 1990). The third study reported a beneficial effect of IVIG in adolescent patients, but a fourth trial reported that IVIG was ineffective (Rowe, 1997; Vollmer-Conna et al., 1997). Despite these conflicting results from clinical trials, some authors believe that IVIG presents a potentially curative treatment for a proportion of patients with ME/CFS and that further randomized controlled trials should be conducted with urgency, especially since many patients with Long COVID met the criteria for ME/CFS (Brownlie and Speight, 2021).

More recently, in a case–control study of patients with post-COVID SFN who had comorbid ME/CFS, IVIG administered to nine patients resulted in decreased allodynia and neuropathic symptoms compared to patients who were not treated with IVIG (McAlpine et al., 2024). Subcutaneous low-dose immunoglobulin therapy has also been shown to be effective in 17 patients with ME/CFS (Sjogren et al., 2024). In a cohort of 20 patients, immunoadsorption was used to remove select immunoglobulins and autoantibodies from plasma, which led to symptomatic improvement in some patients (Stein et al., 2025). Further research involving more robust, controlled study designs with larger sample sizes is needed to elucidate the efficacy of these immunologic therapies for the treatment of ME/CFS.

### 3.4 Potential immunologic therapies for POTS, other common autonomic disorders, and Long COVID

Since POTS, OCADs, and Long COVID have been increasingly linked to autoimmunity and immune system dysregulation, new and repurposed immunologic therapies present a potentially effective treatment option and should be explored in future clinical trials. These therapies may be used either as a last resort in patients who failed standard non-pharmacologic and pharmacologic therapies or as a first-line treatment in patients with POTS and OCADs of suspected autoimmune or inflammatory etiologies, or comorbid SFN, UCTD, and other systemic autoimmune disorders. Many immunologic therapies have already been approved for other indications that could have the potential to treat POTS and OCADs, including immunoglobulin, plasmapheresis, immunoadsorption, corticosteroids, hydroxychloroquine, mycophenolate, azathioprine, methotrexate, monoclonal antibody treatments, and various receptor inhibitors (**Table 4**). Availability and accessibility of these immunotherapies to patients with POTS, OCADs, and Long COVID may present a potentially effective treatment option and prevent future disability incurred as a result of progressive disease course.

**Table 4:** Potential immunotherapies for clinical trial consideration in POTS and OCADs.

Immunotherapy	Mechanism of action	FDA-approved indications
Immunoglobulin (IV or SC) (Immune globulins, 2023)	Antagonism of IgG antibody Fc receptors	- Primary humoral immunodeficiency - Idiopathic thrombocytopenic purpura - CIDP, acute inflammatory demyelinating polyneuropathy (AIDP), MMN, and other neurologic disorders
Plasmapheresis * (Sergent and Ashurst, 2025)	Extracorporeal filtration or exchange of blood plasma	- Guillain–Barré syndrome - AIDP and CIDP - Myasthenia gravis - NMDA receptor antibody encephalitis - Paraproteinemic demyelinating neuropathy - Progressive multifocal leukoencephalopathy associated with natalizumab - Thrombotic thrombocytopenic purpura - Wilson’s disease
Immunoadsorption ** (Prosorba Column Receives FDA Approval for Rheumatoid arthritis treatment, 1999-2025; Report no. H970004A, 1998)	Extracorporeal filtration and removal of IgG antibodies and IgG-bound immune complexes from blood plasma	- Rheumatoid arthritis - Hemophilia A and B
Corticosteroids (Drug Approval Package: Rayos (prednisone) delayed release ta blet 1 mg, 2 mg, 5 mg, 2013) - Methylprednisolone - Prednisone - Hydrocortisone	Synthetic or naturally occurring analogs of adrenal corticosteroids	- Many indications
Hydroxychloroquine (Jorge et al., 2018; Cabral et al., 2019; Coronavirus (COVID-19) update: FDA revokes emergency use authorization for chloroquine and hydroxychloroquine, 2020)	Derivative of 4-aminoquinoline	- Rheumatoid arthritis - Systemic lupus erythematosus - Chronic discoid lupus erythematosus - Malaria
Mycophenolate mofetil (Sollinger, 1995; Vermersch et al., 2005)	Uncompetitive, reversible inosine monophosphate dehydrogenase (IMPDH) inhibitor	- Neuroimmune disorders - Prophylaxis of organ rejection in allogeneic kidney, heart, or liver transplants
Azathioprine (Anstey et al., 2004; Ladriere, 2013)	Purine analog, derivative of 6-mercaptopurine (6-MP) and thioguanine (6-TGN)	- Neuroimmune disorders - Prophylaxis of renal homotransplantation rejection - Rheumatoid arthritis
Methotrexate (Ham et al., 2020; Fraenkel et al., 2021; Hsieh and Tsai, 2024; Hanoodi and Mittal, 2025)	Antagonist of dihydrofolic acid reductase (DHFR)	- Rheumatoid arthritis - Severe psoriasis - Polyarticular juvenile idiopathic arthritis - Cancer
Rituximab (Delate et al., 2020)	Monoclonal antibody against CD20 antigens on pre-B and mature B lymphocytes	- Neuroimmune disorders - Rheumatoid arthritis - Granulomatosis with polyangiitis - Non-Hodgkin’s lymphoma - Chronic lymphocytic leukemia - Pemphigus vulgaris
Adalimumab (Traczewski and Rudnicka, 2008; LiverTox: clinical and research information on drug-induced liver injury, 2012)	Antagonist of tumor necrosis factor-alpha (TNF-alpha) cell surface receptors for p55 and p75	- Rheumatoid arthritis - Juvenile idiopathic arthritis - Psoriatic arthritis and plaque psoriasis - Ankylosing spondylitis - Crohn’s disease and ulcerative colitis - Uveitis
Infliximab (Maini et al., 1999; Pola et al., 2012; Lahad and Weiss, 2015; Fatima et al., 2025)	Antagonist of all tumor necrosis factor-alpha (TNF-alpha) receptors	- Rheumatoid arthritis - Ankylosing spondylitis - Psoriatic arthritis and plaque psoriasis - Crohn’s disease and ulcerative colitis
Imatinib (Inc. A, 2019)	Tyrosine kinase inhibitor (TKI)	- Newly diagnosed Philadelphia chromosome-positive chronic myeloid leukemia - Philadelphia chromosome-positive acute lymphoblastic leukemia - Myelodysplastic/myeloproliferative diseases - Aggressive systemic mastocytosis
Tocilizumab (Syngle et al., 2015; Stone et al., 2017; Finzel et al., 2019; Brunner et al., 2021; Investigators et al., 2021; Salama et al., 2021)	Antagonist of soluble and membrane-bound interleukin-6 (IL-6) receptor	- Rheumatoid arthritis - Polyarticular juvenile idiopathic arthritis - Systemic juvenile idiopathic arthritis - Giant cell arteritis - Coronavirus disease 2019 in hospitalized patients.
Omalizumab (Nowak, 2006; Maurer et al., 2013; Kumar and Zito, 2025)	Antagonist of IgE antibody	- Asthma - Chronic rhinosinusitis with nasal polyps - Chronic spontaneous urticaria

POTS, postural orthostatic tachycardia syndrome; OCADs, other common autonomic disorders; IV, intravenous; SC, subcutaneous; CIDP, chronic inflammatory demyelinating polyneuropathy.

* FDA regulates devices and procedures related to TPE, but not their use in particular conditions.

** FDA regulates devices and procedures related to immunoadsorption, but they granted two specific approvals for its intended use in a medical condition.

## 4 Future direction

Although immunomodulating therapies appear to be beneficial in at least a subset of patients with POTS and OCADs, the next step is to invest in large, multicenter, placebo-controlled trials of immunoglobulin, plasmapheresis, intermittent corticosteroids, and other repurposed immunologic therapies. However, these trials may be more difficult to execute than similar trials for patients with immune-mediated peripheral neuropathies, multiple sclerosis, myasthenia gravis, and other autoimmune disorders. The reasons for these complexities are multifaceted. First, the heterogeneity of the patient population, diverse pathophysiology and autoantibodies, and a lack of a precise unifying biomarker underlying POTS and dysautonomia in general can make it difficult to interpret and generalize the outcomes. Second, the 30-bpm heart rate elevation as a diagnostic criterion for POTS may not be a good marker to assess treatment outcome, as this change in heart rate is highly variable and imprecise. Moreover, there is a lack of established inclusion criteria for patients with presumed autoimmune POTS. Additionally, comorbidity with small fiber neuropathy, UCTD, and autonomic neuropathy, which are predominantly driven by autoimmune and inflammatory etiologies, needs to be considered. Furthermore, the effect of saline and albumin as comparators needs to be examined, as these agents may not be truly placebo and may have significant blood volume and some immunologic effects (Chemali et al., 2024). Another difficulty is the high prevalence of patients with allergies and sensitivities to medications, excipients, and preservatives among patients with POTS; therefore, patients may require individualized and modified trial protocols. Immunotherapy dose, duration, and cross-over timelines also need to be evaluated, given that at least 3–6 months of treatment may be required to see the full effect and that at least 6 months may be needed for the effect of immunotherapy to dissipate. Moreover, the optimal timing of immunotherapy initiation relative to disease onset needs to be determined. It is possible that starting immunotherapy sooner rather than later in the disease course would yield better efficacy and treatment outcomes than starting it at any point in the disease course. Finally, validated questionnaires to assess autonomic symptom burden, fatigue, functional abilities, and quality of life should be used as primary outcomes, and objective heart rate and blood pressure responses should be used as secondary outcomes because there is a high rate of discrepancy and variability between symptom severity and vital signs. Despite these challenges, however, we believe that conducting large, well-designed clinical trials of immunotherapies is a priority for patients with POTS and OCADs, including those with post-COVID onset.

## 5 Conclusion

Combining the limited data outlined in this review, the current and future clinical trials, and our clinical experience, we conclude that immunologic therapies present an important and, potentially, very effective therapeutic option for patients with POTS, OCADs, and Long COVID. To this end, we believe that patients with severe POTS, OCADs, and Long COVID should have access to a variety of therapeutic options involving immunomodulation, including a 3–6-month trial of IVIG, SCIG, or plasmapheresis—therapies that are already available to patients with demyelinating neuropathies, autonomic neuropathy, autoimmune autonomic ganglionopathy, and other neurologic and autoimmune disorders.
